# Structure Elucidation of New Acetylated Saponins, Lessoniosides A, B, C, D, and E, and Non-Acetylated Saponins, Lessoniosides F and G, from the Viscera of the Sea Cucumber *Holothuria lessoni*

**DOI:** 10.3390/md13010597

**Published:** 2015-01-16

**Authors:** Yadollah Bahrami, Christopher M. M. Franco

**Affiliations:** 1 Medical Biotechnology, Flinders Medical Science and Technology, School of Medicine, Flinders University, Adelaide SA 5042, Australia; E-Mail: yadollah.bahrami@flinders.edu.au; 2 Centre for Marine Bioproducts Development, Flinders University, Adelaide SA 5042, Australia; 3 Australian Seafood Cooperative Research Centre, Mark Oliphant Building, Science Park, Adelaide SA 5042, Australia; 4 Medical Biology Research Center, Kermanshah University of Medical Sciences, Kermanshah 6714415185, Iran

**Keywords:** sea cucumber, viscera, saponins, mass spectrometry, MALDI, ESI, HPCPC, triterpene glycosides, structure elucidation, bioactive compounds, marine invertebrate, Echinodermata, holothurian

## Abstract

Sea cucumbers produce numerous compounds with a wide range of chemical structural diversity. Among these, saponins are the most diverse and include sulfated, non-sulfated, acetylated and methylated congeners with different aglycone and sugar moieties. In this study, MALDI and ESI tandem mass spectrometry, in the positive ion mode, were used to elucidate the structure of new saponins extracted from the viscera of *H. lessoni*. Fragmentation of the aglycone provided structural information on the presence of the acetyl group. The presence of the *O*-acetyl group was confirmed by observing the mass transition of 60 u corresponding to the loss of a molecule of acetic acid. Ion fingerprints from the glycosidic cleavage provided information on the mass of the aglycone (core), and the sequence and type of monosaccharides that constitute the sugar moiety. The tandem mass spectra of the saponin precursor ions [M + Na]^+^ provided a wealth of detailed structural information on the glycosidic bond cleavages. As a result, and in conjunction with existing literature, we characterized the structure of five new acetylated saponins, Lessoniosides A–E, along with two non-acetylated saponins Lessoniosides F and G at *m/z* 1477.7, which are promising candidates for future drug development. The presented strategy allows a rapid, reliable and complete analysis of native saponins.

## 1. Introduction

Sea cucumbers belonging to the class *Holothuroidea* of the *Echinodermata* phylum are marine invertebrates that produce a range of compounds that have the potential to be used in agriculture, and as pharmaceuticals, nutraceuticals and cosmeceuticals [1,2].

Saponins are the most important characteristic and abundant secondary metabolites in this species [3]. Sea cucumber saponins exert a wide range of medicinal and pharmacological properties. Saponins are also the main bioactive compounds in many plant drugs and folk medicines, especially in the Orient.

Although sea cucumber saponins share common saponin features, their aglycones, also called sapogenins or genins, are significantly different from those reported in the plant kingdom [1]. These amphipathic compounds generally possess a triterpene or steroid backbone or aglycone (hydrophilic, lipid-soluble) connected glycosidically to a saccharide moiety (hydrophilic, water-soluble) [3,4,5]. Saponins are also produced by other marine organisms including asteroids [6], which also belongs to the phylum *Echinodermata,* and sponges of the phylum *Porifera* [7]. Sea cucumbers saponins are usually triterpene glycosides (derived from lanostane) [3] while those from starfish are steroid glycosides [6]. The sugar moieties mainly consist of d-xylose (Xyl), d-quinovose (Qui), 3*-O-*methyl-d-glucose (MeGlc), 3*-O*-methyl-d-xylose (MeXyl) and d-glucose (Glc), and sometimes 3*-O*-methyl-d-quinovose, 3-*O*-methyl-d-glucuronic acid and 6*-O*-acetyl-d-glucose. In the oligosaccharide chain, the first monosaccharide unit is always a xylose, whereas 3-*O*-methylglucose and/or 3-*O*-methylxylose are always the terminal sugars. The presence of two quinovose residues in a carbohydrate moiety is unique for sea cucumber and starfish glycosides.

There are more than 700 triterpene glycosides in various species of sea cucumbers [3,5,8,9,10,11,12,13,14,15,16,17], which are classified into four main structural categories based on their aglycone moieties: three holostane types containing a (1) 3β-hydroxyholost-9(11)-ene aglycone skeleton; (2) a 3β-hydroxyholost-7-ene skeleton and (3) an aglycone moiety different to other two holostane type aglycones, and a nonholostane aglycone [5,12,17,18]. The majority of saponins belong to the holostane type group [3,12,13,19]. Most sea cucumber saponins comprise of a lanostane-3β-ol type aglycone with a γ-18 (20)-lactone in the d-ring of tetracyclic triterpene (3β,20*S*-dihydroxy-5α-lanostano-18,20-lactone) [5], sometimes containing shortened side chains; the glycone contains up to six monosaccharide units covalently connected to C-3 of the aglycone.

In sea cucumbers, the sugar residue has only one branch [13], whereas plant saponins may contain one, two or three saccharide chains, with a few having an acyl group bound to the sugar moiety [20]. One of the most noteworthy characteristics of many of the saponins from marine organisms is the sulfation of aglycone or sugar moieties [1], and in sea cucumbers the sulfation of one or more of Xyl, Glc, MeGlc and Qui residues have been reported [3,11]. Most of them are mono-sulfated glycosides with few occurrences of di- and tri-sulfated glycosides [13,17]. Another structural feature that has been found only in this series of aglycones is the presence of an acetoxyl group at C-16 and/ or in the lateral side of the aglycone (C-22 or C-23 and/or C-25). The other structural feature is the presence of a 12α-hydroxy group in the aglycone unit of saponins in the genus *Holothuria*; however some contain two hydroxy groups at positions 12α and 17α of the holostanol skeleton. Several triterpene glycosides (such as Holothurinoside X, Fuscocinerosides B and Scabraside B) isolated from the sea cucumber *Holothuria lessoni* contain a carbonyl group in the lateral chain [3,12]. The majority of saponins from Aspidochirotida sea cucumbers contain the 9(11)-double bond in their aglycone moiety, but most of the glycosides isolated from *Holothuria* are ∆^9,11^-glycosides.

Recently, a series of unusual non-holostane triterpene glycoside have been reported from sea cucumbers, belonging to order *Dendrochirotida*, which have a shortened side chain, and no lactone function. So far, only nine non-holostane acetylated saponins including Kurilosides A and C, Psolusoside B, Frondoside C, Cucumariosides A_2_-7, A_2_-8, A_8_, A_9_ and Koreoside A have been reported from the class *Holothuroidea*.

Similarities in structure of saponin glycosides leads to difficulties in purification, and this vitiates the complete structure elucidation of these molecules (especially isomers). High Performance Centrifugal Partition Chromatography (HPCPC) is effective in separating polar compounds and was employed successfully in obtaining purified saponins in this study.

The viscera of an Australian sea cucumber *Holothuria lessoni* (golden sandfish) [21] was selected as a source of saponins because we hypothesized that the internal organs contain high levels of compounds as the viscera are expelled from the sea cucumber in order to repel other sea animals. In relation to internal organs, the saponin content of the cuvierian tubules of *Holothuria* were found to be higher than the body wall on a weight basis [22,23]. We have recently reported [3,12] new saponins within the viscera of *H. lessoni*, and in this paper we present five new acetylated saponins and two related new non-acetylated isomers identified from the viscera using mass spectrometry.

Nuclear magnetic resonance (NMR) spectroscopy can provide extensive structural information for saponins, however larger quantities of high-purity samples are generally needed. This is complicated with fractions if the NMR signals overlap, making their assignments more difficult. Moreover, the measurement of the absolute configuration of the sugar moieties of a saponin cannot be completely solved by NMR methods alone [24]. Matrix-assisted laser desorption/ionization time-of-flight mass spectrometry (MALDI-ToF/MS) and electrospray ionization mass spectrometry (ESI-MS) techniques have become the preferred techniques for analyses of saponins. Mass spectrometry provides a highly sensitive platform for the analyses of saponin structures by generating product ions by the cleavage of the glycosidic bond.

Several studies reported that individual species have specific saponin congeners. However some congeners are common among different species. Even if the diversity is great, saponins from closely related species still retain the same molecular motif [11,25] and this property of saponins can be utilized for their taxonomic classification. Because of their internal and external roles the molecular structure of these compounds was most likely to be conserved within the species.

Mass spectrometry has a long history in the structure elucidation of saponins in both negative and positive ion modes. It has been extensively used to determine the molecular weight and the structure of the native aglycones as well as the glycosidic linkages in the oligosaccharide chain without degradation of the glycosides. Knowledge of the chemical structure of compounds is very important to determine the specific correlation between the structure and their molecular and biological mechanism(s) of actions. We expect that the results of this project will transform the value of the viscera of sea cucumbers into sources of high value products, important to human health and industry.

## 2. Results and Discussion

We reported the isolation and purification of several saponins from the viscera of sea cucumber species, *H. lessoni*, using ethanolic extraction, followed by solvent partition, then HPCPC. The extraction and purification procedures and the mass spectrometry analyses were described in detail in our previous publications [3,12].

The appropriate HPCPC fractions were pooled, based on their similar Rf values when run on thin-layer chromatography (TLC), and concentrated to dryness. Sodium ions were introduced to the samples before conducting the MS analysis, ensuring all saponins observed in the positive ion mode were predominantly singly charged sodium adducts [M + Na]^+^; triterpene glycosides have a high affinity to alkali cations. The prominence of [M + Na]^+^ also facilitated the analysis of saponins in mixtures or fractions. The saponin profile of each HPCPC fraction was then revealed by MALDI MS and ESI-MS [3,12]. MS^2^ analyses identified key diagnostic ions produced by cleavage of the glycosidic bond including oligosaccharide and monosaccharide fragments [3,12,26]. Other visible peaks and fragments detected corresponded to the loss of other neutral moieties such as CO_2_, H_2_O or CO_2_ coupled with H_2_O.

### 2.1. Structure Determination of Saponins by ESI-MS

ESI-MS*^n^* is a very effective and powerful technique to distinguish isomeric saponins as they generate different MS*^n^* fragmentation profiles [27,28]. All saponin ions perceived in the ESI-MS spectrum of the HPCPC fractions were also analyzed by ESI-MS^2^ in the positive ion mode. Previous MS^2^ studies on HPCPC fractions 12, 14, 15 [3], 17, 18, 20 and 22 [12] obtained from the butanolic extract of viscera of sea cucumber *H. lessoni* yielded a number of new saponins. This analysis of fraction 18 gave complex spectra representing several saponin classes, also confirmed the presence of saponins reported in the literature and identified new saponin congeners (Supplementary Figure S1, and Figure 1 of [12]). Fifteen major peaks were detected which corresponded to several known triterpene compounds (as summarized in Table 1 of [12]), including Holothurinosides C/C_1_, Desholothurin A_1_ and Desholothurin A (synonymous with Nobiliside 2a), Holothurinoside J_1_, Fuscocinerosides B/C or Scabraside A or 24-dehydroechinoside A and Holothurin E, Holothurin A, Holothurinosides E/E_1_/O/P, Holothurinoside M, Holothurinosides A/A_1_/R/R_1_/S/Q, Holothurinoside N, Holothurinoside I and Holothurinoside K_1_ in addition to several new saponins [3,12]. The spectrum displays one dominant peak at *m/z* 1477.7, which corresponds to unidentified (new) saponins, with elemental compositions of C_68_H_110_O_33_, C_66_H_102_O_35_ and C_66_H_118_O_34_.

**Figure 1:**
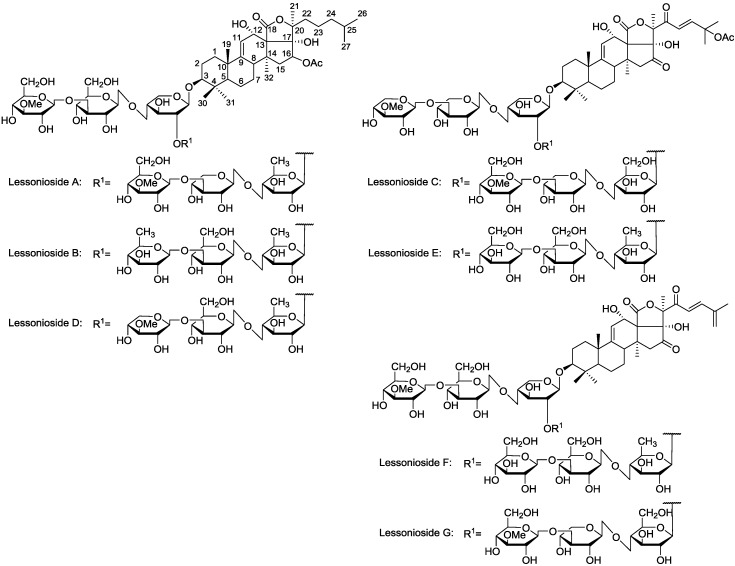
The structures of the new acetylated saponins in the viscera of *H. lessoni*, Lessoniosides (**A**–**E**) along with the non-acetylated Lessoniosides (**F**–**G**) compounds are described in this figure.

This analysis revealed that HPCPC Fraction 18 contains several saponin congeners showing that the absolute purification of the saponins was not possible within a single HPCPC run with these closely related compounds.

### 2.2. Structure Identification of Saponins by MALDI-MS

Similar to the ESI-MS, the MALDI MS of the isobutanol-enriched saponin extract obtained from the viscera of the *H. lessoni* revealed the presence of at least 75 saponin congeners, including 39 new sulfated, non-sulfated and acetylated triterpene glycosides, and 36 congeners which were previously reported in other holothurians [3].

To elucidate the chemical structure of saponins based on the MS^2^ spectra, as described previously [3,12], precursor ions were selected, fragmented and fragmentation profiles built. The molecular structures of the saponins were determined by the identification of the mass transitions between the successive collision-induced fragmentation peaks on the basis of the accurate mass of the individual sugar components.

Based on the literature, MeGlc and MeXyl are always terminal sugars and Xyl is always the first sugar, which is bound to C-3 of the aglycone. Further, the exact mass of each sugar, such as MeGlc = 176 Da, Glc = 162 Da, Xyl = 132 Da, Qui = 146 Da, and the determination of the mass transitions between the peaks on the basis of the accurate mass of the individual sugar moieties, and mass and sequence of the key diagnostic peaks helped us build the sequence of these sugar moieties. Using this strategy the structure of seven new triterpene glycosides from *H. lessoni* with an *m/z* value of 1477.7 from HPCPC fraction 18 were characterized.

The chemical structures of the new acetylated saponins from the viscera of *H. lessoni* are illustrated in Figure 1. Lessoniosides A, B, C, D and E are the only published examples of glycosides from *H. lessoni* containing the side chain of the acetoxy group in their aglycone moieties. We now provide an account of the structure elucidation of these saponins using this approach.

#### MALDI-MS^2^ Analysis of Saponins

**Figure 2:**
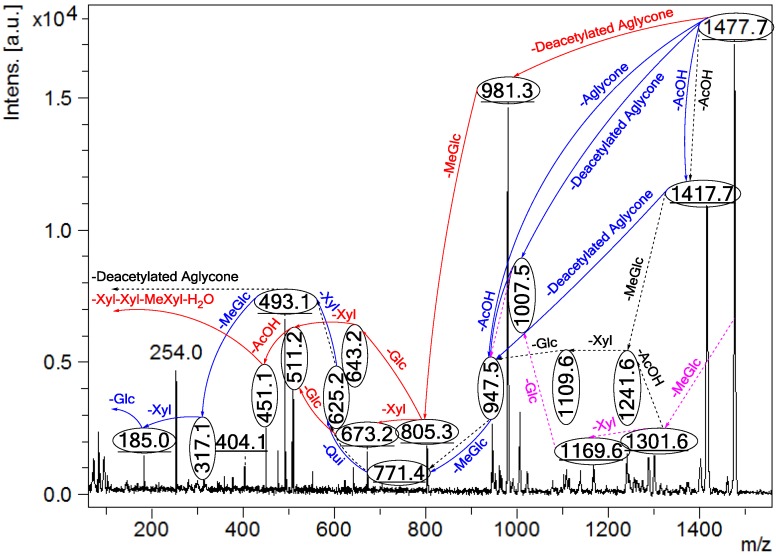
Positive tandem MALDI (matrix-assisted laser desorption/ionization) spectrum analyses of the precursor ion (saponin) detected at *m/z* 1477.7. The MS^2^ fragmentation profile of the ion at *m/z* 1477.7. Figure shows the collision-induced fragmentation of parent ions at *m/z* 1477.7. The full and dotted arrows show the possible fragmentation pathways of this ion using CID (collision-induced dissociation). The blue arrows show the fragmentation of the isomeric congeners Lessonioside A where the red arrows indicate the decomposition patterns of Lessonioside C. These analyses revealed that this ion corresponds to isomeric compounds.

Saponin ion peaks were further analyzed using MS^2^ fingerprints generated with the collision-induced dissociation (CID) from their respective glycan structures. The techniques used are also able to distinguish the structural differences among the isomers following HPCPC separation. As a typical example, the MALDI-MS^2^ fingerprints for the ion detected at *m/z* 1477.7 (triterpene glycoside) are shown in Figure 2. The schematic fragmentation of Lessonioside A as a representative is shown in Supplementary Figure S2. The fragmentation pattern of the sodiated compound at *m/z* 1477.7 in consecutive MS experiments is discussed in detail below for stepwise elucidation of the molecular structure of these compounds.

**Figure 3:**
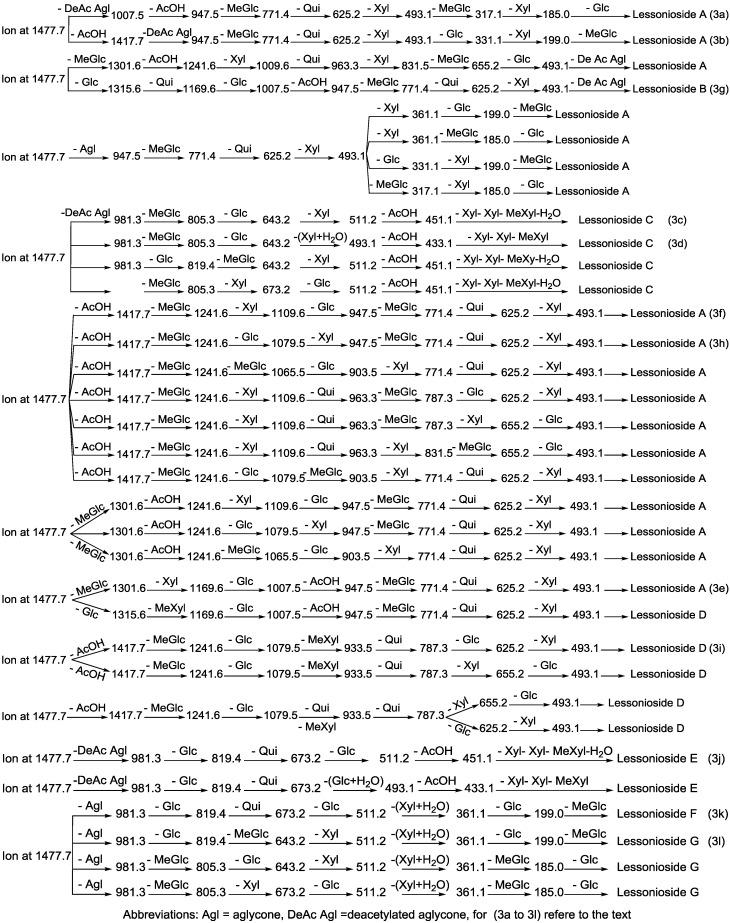
The schematic diagram of the proposed isomeric structures of the ion at *m/z* 1477.7. This figure indicates the comprehensive feasible fragmentation pathways of the isomeric acetylated, Lessoniosides (**A**–**E**), and non-acetylated, Lessoniosides (**F**–**G**), triterpene glycosides generated from the ion at *m/z* 1477.7.

CID activates three feasible independent fragmentation pathways of cationized parent ions shown in full and dotted arrows. First, as described in Figure 2, the consecutive losses of the deacetylated aglycone, acetic acid (AcOH), 3-*O*-methyl-d-glucose (MeGlc), d-quinovose (Qui), d-xylose (Xyl), MeGlc and Xyl residues (blue arrows) followed by d-glucose (Glc) yielded ion fragments at *m/z* 1007.5, 947.5, 771.4, 625.2, 493.1, 317.1 and 185.0 (Figure 3a), respectively, in one of the new isomers for which we propose the name Lessonioside A. The loss of aglycone (Agl) generated the ion at *m/z* 947, corresponding to the complete sugar moiety. The ion at *m/z* 493.1 corresponds to the diagnostic sugar reside [MeGlc-Glc-Xyl + Na]^+^. Further, the sequential losses of Glc and Xyl units from this key diagnostic peak (*m/z* 493.1) generated ions at *m/z* 331.1 and 199.0 (Figure 3b).

With another isomer, the consecutive losses of the deacetylated aglycone, MeGlc, Glc, Xyl and AcOH followed by the hydrated three sugar units (red arrows) produced ions at *m/z* 981.3, 805.3, 643.2, 511.2 and 451.1, respectively, (Figure 3c) revealed the structure of a second new saponin, which we named Lessonioside C. Further, the consecutive losses of the deacetylated aglycone, MeGlc, Glc, Xyl (at a terminal position) and an acetyl group from the parent ion generated the fragment ions at *m/z* 981.3, 805.3, 643.2, 493.1 and 433.1, (Figure 3d) respectively, confirming the structure of Lessonioside C.

Secondly, the decomposition of the parent ion can also be triggered by the sequential loss of sugar moiety namely MeGlc, Xyl, Glc, AcOH, MeGlc, Qui and Xyl followed by the deacetylated aglycone residue which generated daughter ions at *m/z* 1301.6, 1169.6, 1007.5, 947.5, 771.4, 625.2, and 493.1, respectively (Figure 3e). This sequence of fragmentation confirms the structure of new saponin, Lessonioside A. In this case, the ions at *m/z* 493.1 correspond to the sodiated deacetylated aglycone moiety (*m/z* value of 470).

The third viable pathway is elicited by the initial loss of an acetoxy group. In the case of Lessonioside A this initial loss (−60) is followed by the sequential loss of the sugars (including the diagnostic MeGlc-Glc-Xyl) to yield the key diagnostic DeAc Agl ion (*m/z* 493.1) (Figure 3f). In addition the sequential losses of the MeGlc (317.1) and Xyl (*m/z* 185.0) followed by Glc further confirmed the structure of the new isomer, Lessonioside A.

As was observed with the MALDI-MS^2^ this saponin possesses the common *m/z* 493.1 key signal diagnostic of both the sugar moiety [MeGlc-Glc-Xyl + Na]^+^ and the DeAc Agl moiety [C_32_H_50_O_6_ − AcOH + Na]^+^. This is consistent with previous findings for the MS^2^ of sea cucumber saponins [12]. The ion 493.1 is also observed in Lessonioside C, however, this is formed as a result of a loss of DeAc Agl and of the sugar moiety MeGlc-Glc-Xyl (511) and H_2_O (493). The ion at 643 yields ions at 511 and 493 by the loss of Xyl and Xyl + H_2_O, respectively. These ions are recognized as the key diagnostic fragments in triterpenoid saponins.

These MS^2^ analyses using both MALDI and ESI modes allowed the establishment of connectivities of the sugar residues and thus permit the assignment of the peaks. For example, the MALDI-MS^2^ of the parent ion showed fragments at *m/z* 1417.7 [M + Na − AcOH]^+^ which suggested the presence of an acetyl moiety and the innate sugar component at *m/z* 947.5 [M + Na − Agl]^+^, an observation that was confirmed by ESI- MS^2^ in the positive ion mode. The MALDI mass spectrum showed evidence of the presence of the acetoxy group in the aglycone.

### 2.3. Key Diagnostic Sugar Residues in the Sea Cucumber Saponins

Characterization of common key fragments expedited the structure elucidation of new and reported saponins. Tandem mass spectrometry analysis of saponins showed the presence of several diagnostic key fragments corresponding to certain common structural element of saponins as summarized in Table 1. Here we report a new diagnostic key fragment at *m/z* 643 corresponding to the sodiated hydrated sugar residue MeGlc-Glc-Xyl-Xyl.

**Table 1:** Key diagnostic ions in the MS^2^ of the holothurians saponins. It has been adapted from [12] and modified.

Diagnostic Ions in CID Spectra of Saponins [M + Na]^+^				
*m/z* Signals (Da)				
	493	507	643 or 625	639 or 657
Chemical signatures	MeGlc-Glc-Xyl + Na	MeGlc-Glc-Qui + Na	MeGlc-Glc-Xyl-Xyl + Na = 625 MeGlc-Glc-Xyl-Xyl + H_2_O + Na = 643	MeGlc-Glc-Qui-Xyl + Na = 639 MeGlc-Glc-Qui-Xyl + H_2_O + Na = 657

The structures of sugar components of saponins were established by the identification of these diagnostic ions produced by tandem mass spectrometry. Observing these oligosaccharide moieties (*m/z* 493 and/or 507 and/or 511 (493 + H_2_O) and/or 523 and/or 643 and/or 657) simplified the characterization of the saponin structure.

### 2.4. Elucidation of the Saponin Structures by ESI-MS^2^

**Figure 4:**
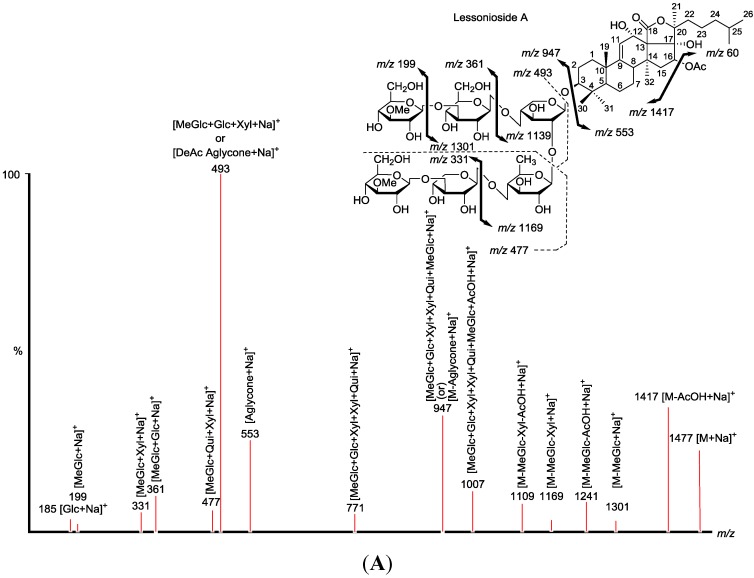

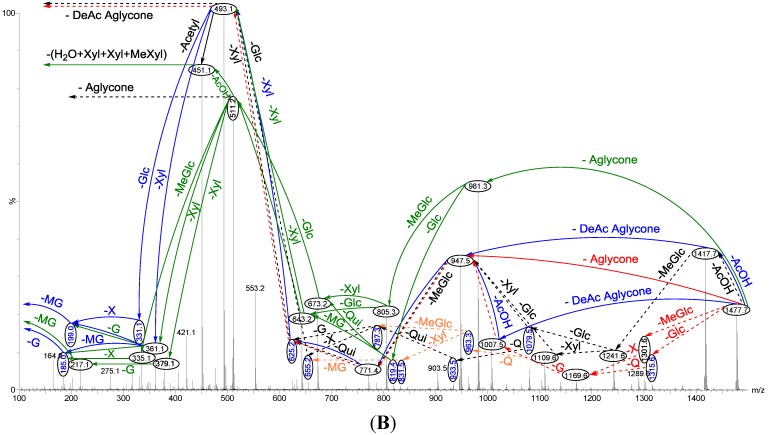
Positive ion mode ESI-MS^2^ spectrum of acetylated saponins detected at *m/z* 1477.7 from Fraction 18. The schematic fragmentation of Lessonioside A as a representative (**A**), and the complete ESI-MS^2^ fragmentation profile of the ion at *m/z* 1477.7 (**B**). Spectrum (**B**) shows the presence of two different aglycones in the isomeric saponins. Full and dotted arrows illustrate the three main feasible fragmentation pathways. The blue arrows show the decomposition of the isomeric congeners Lessoniosides A, B and D where the green arrows indicate the fragmentation patterns of Lessoniosides C, E, F and G. The ion at *m/z* 451.1 corresponds to the hydrated three sugar units [Xyl-Xyl-MeXyl + H_2_O + Na].

ESI-MS^2^ was carried out using CID, creating ion fragments from the precursor ions (Figure 4), and was applied to differentiate the structure of isomeric saponins as described by Song *et al.* [27]. The schematic fragmentation of Lessonioside A, as a representative, and the stepwise structure elucidation of the ion at *m/z* 1477.7 is shown in Figure 4 corroborates results from the MALDI-MS^2^.

#### 2.4.1. ESI- MS^2^ Analyses of Ion at *m/z* 1477.7

Tandem MS analyses revealed the presence of two different peaks with *m/z* value of 947.5 and 981.3, corresponding to the losses of different aglycone moieties with *m/z* values of 530 and 496, respectively, confirming the presence of chemical structural isomers. Further this MS^2^ analysis also distinguished the presence of an acetoxy group in both isomer types.

Similar to sulfated compounds, after collisional activation, the parent ions are subjected to three independent dissociation pathways shown using full and dotted arrows (Figure 4). First, the consecutive losses of the deacetylated aglycone, acetoxy group, MeGlc, Qui, Xyl, Xyl and MeGlc residues (blue arrows) followed by Glc afford product ions as shown in Figure 4 confirmed the structure of Lessonioside A. Therefore, in this case, the ions at *m/z* 493.1 correspond to the sodiated key diagnostic sugar residue; [MeGlc-Glc-Xyl + Na]^+^.

Secondly, the decomposition of the parent ion could also be triggered by the loss of sugar moieties followed by the deacetylated aglycone residue which generated daughter ions as shown in Figure 3e confirming once more the structure of Lessonioside A. It is clear that the ions at *m/z* 493.1 correspond to the sodiated deacetylated aglycone moiety (*m/z* value of 470). Alternatively, ions corresponding to the sequential losses of Glc, Qui, Glc, AcOH, MeGlc, Qui, and Xyl (red dotted arrows) were detected in Figure 3g indicating the presence of another isomer, Lessonioside B, which possesses two Qui units. The presence of two Qui in the carbohydrate chain of sea cucumber glycoside is a very rare characteristic.

Finally, the fragmentation of the parent ions can also be initiated with the loss of the acetoxy group. The consecutive losses of the acetic acid (AcOH) and the deacetylated aglycone unit followed by the sequential losses of the sugar moiety (Figure 3b) further confirmed the structure of Lessonioside A. Alternatively, the decomposition of the deacetylated saponins can be accomplished by the sequential losses of monosaccharides in the sugar chain, namely ion detected at 1417.7 [M − AcOH + Na]^+^ (black dotted arrows, Figure 3h). In this case, the ions at 493.1 corresponds to the DeAc Agl moiety [M − sugar residue − AcOH + Na]^+^. Alternatively, the sequential losses of AcOH and sugars from the parent ions (Figure 3i), afforded daughter ions that assisted in postulating the structure of another new isomer, Lessonioside D. The above evidence suggested that Lessonioside A possesses the same aglycone as Lessoniosides B and D, but differs in the hexasaccharide chain. The complete analyses can be seen in Supplementary Figure S3.

The MALDI-MS^2^ and ESI-MS^2^ analyses for all possible isomers were carried out in a similar manner as described above for Lessoniosides A, B, C and D. A comprehensive list of possible fragmentation patterns based on the MS^2^ ions generated from the ion at *m/z* 1477.7 is shown in Figure 3.

The sugar moiety of Lessonioside A was found to be identical to those of Cladolosides C_1_ and C_2_ isolated from the sea cucumber *Cladolabes schmeltzii* [16], confirming the constituents of the hexasaccharide chain (Figure 1 and Figure 3). The sugar component also had some similarity to those of Violaceuside B isolated from the sea cucumber *Pseudocolochirus violaceus* [29]. This group also stated the ions at *m/z* 625.2 and 493.1 corresponded to [MeGlc + Xyl + Glc + Xyl + Na]^+^ and [MeGlc + Xyl + Glc + Na]^+^, respectively, which confirmed our results. Yayli and associates [30], however, stated the ions at *m/z* 493 and 325, corresponding to [MeGlc-*O*-Xyl-*O*-Qui(*O*)-*O*]^+^ and [MeGlc-*O*-Xyl]^+^, respectively, which are under question. The structure of the aglycone moiety was also very similar to that of Holothurinoside Y [12], the difference being the addition of an acetoxy group at C-16. The assignments of the MS^2^ signals associated with the aglycone moiety Lessoniosides A, B and D showed a close similarity to those reported for 16β-acetoxy-holosta-9-ene-3β,12α,17α-triol, the aglycone of Nobiliside C [*m/z* 715], *m/z* 656 [M − OAc + Na]^+^, isolated from the sea cucumber *Holothuria nobilis* [31].

These saponin congeners identified from Fraction 18 are more conjugated with glycosides compared with the new saponins previously reported in this species [3,12]. Lessoniosides C, D and E possess the same terminal saccharide moiety (MeXyl), which is a rare structural feature among naturally occurring sea cucumber glycoside and has been infrequently reported.

#### 2.4.2. Isomers that Generate the Deacetylated Aglycone at *m/z* 981.3

For other isomers, the loss of the deacetylated aglycone yields a large fragment at *m/z* 981.3. The alternative fragmentation patterns of the sugar residues for Lessoniosides C and E are described in Figure 3.

An interesting peculiarity of Lessonioside C and E is the presence of keto groups at both C-16 and C-22 positions, and 25β-*O*-Ac in the aglycone. This is the third example of the aglycone of sea cucumber glycosides bearing a ketone group at C-22 as Zhang *et al.* [32] and Liu *et al.* [33] stated the presence of a ketone group in Fuscocineroside A and Arguside D, respectively. The structure is characterized by the presence of an oligosaccharide chain composed of six units. The holostane-type aglycone features an endocyclic double bond at position C-9(11) and C-23 and a β-acetoxy group at C-25.

#### 2.4.3. Non-Acetylated Isomeric Congeners

Based on the chemical evidence the structure of Lessoniosides F/G were defined as 16,22-diketo-holosta-9(11)-23(24)-25(27)-triene-3β,12α,17α-triol, which is shown in Figure 1. They are of the lanosterol type featuring the characteristic ring-d-fused γ-lactone (C=O) function and a ∆^9^ double bond. The aglycone of Lessonioside F/G differs from E by the presence of a double bond at ∆^25^ and a loss of the AcO group at C-25 to form an exo double bond, ∆^25^.

On fragmentation these isomers generate ions at 981.3 corresponding to the loss of aglycone. Subsequent losses of the sugar moieties are described in detail in Figure 3 (k and l) and Supplementary Figure S4 for Lessoniosides F and G. These non-acetylated Lessoniosides possess similar aglycones but different sugar moieties. It is notable that all three feasible independent fragmentation pathways might occur simultaneously which generated several different fragmentation sequences.

The molecular weights of the deacetylated aglycone moieties in some of these new saponins (Lessoniosides A, B and D) coincided with those reported for Philinopside B, from the sea cucumber *Pentacta quadrangularis* using ESI-MS by Zhang *et al.* [34], however, the structures are very different, because they are from a different family of sea cucumber, with ring closure of the C-20 side chain.

The cleavage of the *O*-acetyl group of the aglycone residue results in the loss of AcOH (60 Da). However, Song and co-workers [27] noted the neutral losses of CH_2_O (30 Da) and C_2_H_4_O_2_ (60 Da) in cross-ring reactions of the sugar residues. The cross-ring cleavage of the sugar also leads to the loss of C_2_H_4_O_2_ (60 Da).

The losses of H_2_O and CO_2_ or their combination results from cleavage at the glycosidic linkages as noted by Waller and Yamasaki [2]. The losses of CO_2_ (44 Da), H_2_O (18 Da), AcOH (60 Da), Acetyl group (42 Da) and CH_2_O (30 Da) were detected from the spectra which affords different product ions and several peaks were assigned to those molecules. For instance, the ion at *m/z* 1373.7 was generated by the loss of CO_2_ from the deacetylated parent ions (*m/z* 1417.7), or the sequential losses of H_2_O and acetyl molecule from the ions at *m/z* 511.2 generated ions at *m/z* 493.1 and 451.1, respectively. Further the ion at *m/z* 493.1 can be stemmed from the ion at *m/z* 553.2 (sodiated aglycone) by the loss of the acetoxy group. Additionally, the loss of CH_2_O from the ion at *m/z* 451.1 yields the ion at *m/z* 421.1. However, Kelecom and coworkers [35], and Kitagawa and associates [36], stated the latter ion as an aglycone fragment. The complete analyses can be seen in the Supplementary Figure S3.

The MS^2^ analyses of ions at *m/z* 1477.7 revealed a similar fingerprint profile with those reported for Holothurinosides X, Y and Z, in particular in the area of 100 to 600 Da where the signals were coincident with those of ion at *m/z* 1127.6, which show the intrinsic relationship between these saponin congeners [12].

Lessoniosides A, B, C, D and E are the only examples of glycosides from *H. lessoni* containing an acetoxy side chain in their aglycone moieties. Most of the glycosides isolated from sea cucumber *Holothuria* are ∆^9,11^- glycosides. In general, 3β-hydroxyholost-9(ll)-ene based aglycones were characterized in Holothurins isolated from animals of the order *Aspidochirota* [17].

### 2.5. The Structure of Aglycones

This analysis revealed the presence of at least seven different isomers with diverse aglycone and sugar components for the ion at *m/z* 1477.7. The glycosides differ in their aglycone structures or sugar moieties. The mass of the cationized aglycone and the deacetylated aglycone in Lessonioside C and E are 556 Da [M − sugar residue + Na]^+^ and 496 [M − AcOH − sugar residue + Na]^+^_,_ respectively, which are consistent with the mass of the aglycone reported by Elyakov and co-workers [37]. Their MS analyses showed *m/z* 556 [M]^+^, 541 [M − CH_3_]^+^ and 496 [M − CH_3_COO]^+^ for an aglycone moiety. Further, Rothberg and associates noted an aglycone with the same molecular weight having an acetoxy group at the C-23 from *Stichopus chloronotus* [38]. Analysis of the MS data for these isomers and comparison with those published for related saponin aglycones [3,12,31,39] shows that the aglycone part of Lessoniosides are a holostane skeleton featuring hydroxy groups at C-12 and C17. With other isomers, the mass of the cationized aglycone and the deacetylated aglycone was found to be 530 Da and 493 Da, respectively. Other prominent high mass ions *m/z* 1417 [M − AcOH + Na]^+^ and 1241 [M − AcOH − MeGlc + Na]^+^ or 1241 [1301 − AcOH + Na]^+^ provide additional support for the acetate and lactone functions.

The aglycone structure of Lessoniosides C/E appears to be similar to that of Fuscocineroside A reported from the sea cucumber *Holothuria fuscocinerea* [32]. The lateral C-20 side chain of Lessoniosides C and E was found to be similar to Fuscocineroside A [32], and Arguside D [33]. However, they differed from Fuscocineroside A and Arguside D by the addition of a keto at C-16, a C-23 double bond and a 17-OH.

Three of these compounds, Lessoniosides A, B and D have identical holostane aglycones containing an 18(20)-lactone with a 9(11)-double bond and acetoxy group at C-16 and differ from each other in their sugar component. On the other hand, they differ from Lessoniosides C/E in the presence of an acetoxy group at the C-16 (*vs.* keto group in C/E) and absence of a C-22 keto group, a C-23 double bond and a C-25 acetoxy at the lateral chain.

These glycosides have holotoxinogenin, a genin containing 9(11)-double bond and 16-oxidized group in the aglycone. They possess two hydroxy groups at 12α and 17α positions that are characteristic for Aspidochirotid sea cucumber (the family *Holothuriidae*). This aglycone is common for glycosides from many different sea cucumbers [11].

Therefore, elucidation of the aglycone component of the saponins was performed by comparison with published data. Because the new identified compounds clearly have aglycone structures similar to that of other previously characterized compounds we can be confident that it is possible to elucidate the structure of these compounds based on MS analysis alone. However, NMR analysis will be required to confirm the structure of the aglycones and also to ascertain the stereochemistry and linkages of the sugar moieties. Whereas, in the case of a novel compound without any similar previously characterized components, detailed chemical analysis including the application of NMR would be required.

### 2.6. Acetylated Saponins

So far more than 700 triterpene glycosidic saponins have been reported from sea cucumber species of which more than 130 are acetylated saponin. The majority of these are of the holostane type. The known acetylated triterpene glycosides (saponins), isolated from sea cucumbers of the class *Holothuroidea*, possess an acetyl group (acetoxy) in their aglycone residues. In the *Holothuriidae* family, the acetoxy group is either located at C-16 of the aglycone core moiety such as in Arguside F [40] and Nobiliside C [31], or at the C-22, C23 or C-25 of the lateral chain, ie at C-25 of the Pervicosides A and D [40,41]. However, Cucumarioside A_1_-2 is the only example of a triterpene glycoside containing an acetate group at C-6 (6-OAc) of the terminal glucose unit (sugar residue) 6-*O*-acetylglucose [42].

The majority of the acetylated compounds, such as Fuscocineroside A from the sea cucumber *H. fuscocinerea,* contain a sulfate group in their structures [32]. However, the presence of a sulfate group was not observed in these new acetylated saponins from *H. lessoni*.

Acetylated saponins are mainly reported in the family *Cucucmarridae*. However, the presence of acetylated saponins for the genus *Holothuria* is only reported for *H. lessoni* (this work), *H. pervicax*, *H. forskalii*, *H. nobilis*, *H. hilla*, *H. fuscocinerea*, *H. (Microthele) axiloga* and *H. pervicax* [17,31,32,40,41,43,44]. The majority of reported acetylated saponins possess only one acetoxy group in their structure, whereas saponins containing two *O*-acetic groups in their aglycone moieties have also been reported [16].

The presence of 12α and 17α–hydroxy, which are characteristic for glycosides from holothurians belonging to the family *Holothuriidae* (order *Aspidochirotida*), in glycosides of Dendrochirotids confirms parallel and relatively independent character of evolution of glycosides.

Observations from numerous studies confirm that the biological activity of saponins is influenced both by the aglycone and the sugar moiety. In other words there is a close relationship between the chemical structure of saponins and their biological activities. It has been reported that the presence of acetyl groups usually increases cytotoxic potency [45]. Therefore Lessoniosides seem to be potential candidates for anti-cancer drugs development.

## 3. Experimental Section

### 3.1. Sea Cucumber Sample

Twenty sea cucumber samples of *H. lessoni* [21], commonly known as Golden sandfish were collected off Lizard Island (latitude; 14°41′29.46ʺ S, longitude; 145°26′23.33ʺ E), Queensland, Australia on September 2010 [3,12]. The viscera (all internal organs) were separated from the body wall and kept separately in zip-lock plastic bags which were snap-frozen, then transferred to the laboratory and kept at −20 °C until use.

### 3.2. Extraction of Saponins

The saponins were extracted as described previously [3,12]. Briefly, the visceral masses were removed, freeze dried (VirTis, BenchTop K, New York, NY, USA) and pulverized to a fine powder using liquid nitrogen and a mortar and pestle. The pulverized viscera sample was extracted four times with 70% ethanol (EtOH) (400 mL) and filtered using Whatman filter paper (No. 1, Whatman Ltd., Maidstone, England, UK) at room temperature. The extract was concentrated under reduced pressure at 30 °C using a rotary evaporator (Büchi AG, Flawil, Switzerland) to remove the ethanol, and the residual sample was freeze-dried. The dried extract was dissolved in 400 mL of 90% aqueous methanol (MeOH), and partitioned against 400 mL of *n*-hexane twice. The water content of the hydromethanolic phase was then adjusted to 20% (v/v) and then to 40% (v/v) and the solutions partitioned against CH_2_Cl_2_ and CHCl_3_, respectively. The hydromethanolic phase was concentrated and then freeze-dried. The dried powder was solubilized in 10 mL of MilliQ water (18.2 MΩ, Millipore, Bedford, MA, USA) in readiness for chromatographic purification.

### 3.3. Purification of the Extract

The aqueous extract was applied to an Amberlite^®^ XAD-4 column (250 g XAD-4 resin 20–60 mesh; Sigma-Aldrich, MO, USA; 4 × 30 cm column) [3,12], washed extensively with water (1 L) and the saponins eluted sequentially with MeOH (450 mL), acetone (350 mL) and water (250 mL). The MeOH, acetone and water eluates were concentrated, dried, and redissolved in 5 mL of MilliQ water. Finally, the aqueous extract was partitioned with 5 mL isobutanol (v/v). The isobutanolic saponin-enriched fraction was either stored for subsequent mass spectrometry analyses or concentrated to dryness and the components of the extract were further purified by HPCPC. The profile of fractions was also monitored by TLC.

### 3.4. Thin Layer Chromatography (TLC)

Samples were dissolved in 90% or 50% aqueous MeOH and 10 µL were loaded onto silica gel 60 F_254_ aluminum sheets (Merck # 1.05554.0001, Darmstadt, Germany) and developed with the lower phase of CHCl_3_:MeOH:H_2_O (7:13:8) biphasic solvent system [3]. The profile of separated compounds on the TLC plate was visualized by UV light and by spraying with a 15% sulfuric acid in EtOH solution and heating for 15 min at 110 °C until maroon-dark purple spots developed.

### 3.5. High Performance Centrifugal Partition Chromatography (HPCPC or CPC)

The solvent system containing CHCl_3_:MeOH:H_2_O–0.1% HCO_2_H (7:13:8) was mixed vigorously in a separating funnel and allowed to reach hydrostatic equilibration [3,12]. Following the separation of the two-immiscible phase solvent systems, both phases were degassed using a sonicator-degasser (Soniclean Pty Ltd. Adelaide, SA Australia). Then the rotor column of HPCPC™, CPC240 (Ever Seiko Corporation, Tokyo, Japan) was filled with the stationary phase (the aqueous upper phase) in the descending mode at a flow rate of 5 mL min^−1^ by Dual Pump model 214 (Tokyo, Japan), with a revolution speed of 300 rpm. The lower mobile phase was pumped in the descending mode at a flow rate of 1.2 mL min^−1^ with a rotation speed of 900 rpm within 2 h. One hundred and twenty milligrams of isobutanol-enriched saponins mixture was injected into the machine in the descending mode. The chromatogram was developed for 3 hours at 1.2 mL min^−1^ and 900 rpm using the Variable Wavelength UV-VIS Detector S-3702 (Soma optics, Ltd. Tokyo, Japan) and chart recorder (Ross Recorders, Model 202, Topac Inc. Cohasset, MA, USA). The fractions were collected in 3 mL tubes using a Fraction collector. At Fraction 54, the elution mode was switched to ascending mode and the aqueous upper phase was pumped at the same flow rate for 3 h to recover saponins. Fractions were monitored by TLC as described above. Monitoring of the fractions was necessary, as most of the saponins could not be detected by UV due to the lack of a chromophore structure. Fractions were concentrated with nitrogen gas.

### 3.6. Mass Spectrometry

The resultant HPCPC purified polar extracts were further analyzed by MALDI- and ESI-MS to elucidate and characterize the molecular structures of compounds.

#### 3.6.1. MALDI MS

MALDI analysis was carried out using a Bruker Autoflex III Smartbeam (Bruker Daltonik, Bremen, Germany). All MALDI MS equipment, software and consumables were from Bruker Daltonics. The laser (355 nm) had a repetition rate of 200 Hz and operated in the positive reflectron ion mode for MS data over the mass range of 400 to 2200 Da under the control of the Flexcontrol and FlexAnalysis software (V 3.3 build 108). External calibration was performed using the sodium-attached ions from a Polyethylene Glycol of average molecular weight 1000. MS spectra were processed in FlexAnalysis (version 3.3, Bruker Daltonik, Bremen, Germany). MALDI MS^2^ spectra were obtained using the LIFT mode of the Bruker Autoflex III with the aid of CID. The isolated ions were subjected to collision against argon in the collision cell to be fragmented, affording intense product ion signals. For MALDI a laser was used to provide both good signal levels and mass resolution with the laser energy for MS^2^ analysis being generally 25% higher than for MS analysis.

The samples were spotted onto a MALDI stainless steel MPT Anchorchip TM 600/384 target plate. Alpha-cyano-4-hydroxycinnamic acid (CHCA) in acetone/iso-propanol in ratio of 2:1 (15 mg mL^−1^) was used as a matrix to produce gas-phase ions. The matrix solution (1 µL) was placed onto the MALDI target plate and air-dried. Subsequently 1 µL of sample was added to the matrix crystals and air-dried [3,12]. Finally, 1 µL of NaI (Sigma-Aldrich # 383112, St Louis, MI, USA) solution (2 mg/mL in acetonitrile) was applied onto the sample spots. The samples were mixed on the probe surface and dried prior to analysis.

#### 3.6.2. ESI MS

The ESI mass spectra were attained with a Waters Synapt HDMS (Waters, Manchester, UK). Mass spectra were acquired in the positive ion mode with a capillary voltage of 3.0 kV and a sampling cone voltage of 100 V.

The other conditions were as follows: extraction cone voltage, 4.0 V; ion source temperature, 80 °C; desolvation temperature, 350 °C; desolvation gas flow rate, 500 L h^−1^ [3,12]. Data acquisition was performed using a Waters MassLynx (V4.1, Waters Corporation, Milford, CT, USA). Positive ion mass spectra were acquired in the V resolution mode over a mass range of 100–2000 *m/z* using continuum mode acquisition. Mass calibration was performed by infusing sodium iodide solution (2 µg/µL, 1:1 (v/v) water:isopropanol). For accurate mass analysis a lock mass signal from the sodium attached molecular ion of Raffinose (*m/z* 527.1588) was used through the LockSpray source of the Synapt instrument.

MS^2^ spectra were obtained by mass selection of the ion of interest using the quadrupole, fragmentation in the trap cell where argon was used as collision gas. Typical collision energy (Trap) was 50.0 V. Samples were infused at a flow rate of 5 µL/min, if dilution of the sample was required then acetonitrile was used [27]. Chemical structures were determined from fragmentation schemes calculated on tandem mass spectra and from the literature.

## 4. Conclusions

In recent years, there has been a great improvement in the number of MS applications. The tandem MS approach coupled with HPCPC separation revealed the structure of isomeric compounds containing different aglycones and/or sugar residues. Therefore, a creative and sensitive method has been developed for the structure elucidation of triterpene glycosides in sea cucumber and related products using HPCPC and MS. The result showed that this method is a rapid, accurate and reliable technique for the structure determination of triterpene glycosides in sea cucumber extracts.

This study proved the occurrence of both glycoside and cross-ring cleavages in the sugar moieties of sea cucumber saponins. The sequence of monosaccharide units and the presence of an acetoxy group, clearly reflected by the loss of 60 Da from the parent ions, were noted in five of the seven new saponins.

Tandem mass spectrometry data suggested that the most prominent ions generally stemmed from the losses of aglycones and/or the key diagnostic sugar moieties (*m/z* 493, 507, 511, 639, 643 and 625).

Our results also illustrate that some saponins are unique to the species, whilst others are common between multiple species. The MS analysis revealed that individual species possesses a unique saponin pattern in which some congeners are very specific to one species. This feature can be used for the taxonomic classification of sea cucumber species.

Characterization of some of these saponins were easier since their MS^2^ spectra possessed the key diagnostic signal at *m/z* 493, corresponding to the oligosaccharide chain [MeGlc-Glc-Xyl + Na^+^] in addition to the vital peak at *m/z* 643, corresponding to the oligosaccharide moiety [MeGlc-Glc-Xyl-Xyl-H_2_O + Na^+^]. Lessoniosides C, D and E contain 3-*O*-methylxylose as a terminal monosaccharide unit, which is a rare structural feature in sea cucumber triterpene glycosides.

The ion at *m/z* 1477.7 was identified as the major component of the glycoside fraction 18, containing holostane aglycones with 9(11)-double bond and 18(20)-lactone, characteristic for most of the known sea cucumber glycosides. The structures of these glycosides are quite different from those reported in this species. These substances contain aglycones with an oxidized position at C-16, (acetate group or keto group). Lessoniosides have the aglycone unit like that in Holothurinoside Y with an acetoxyl instead of hydrogen at position 16, but another aglycone moiety with the saturated side chain and with an acetoxyl (a 16β-acetate group) instead of a ketone at position 16.

Our results to date highlight the abundance of new saponins in the viscera indicating the viscera as a major source of these compounds with diverse structures. This paper is the first not only to deduce the structure of several new acetylated isomeric saponins, (Lessoniosides A–G) but also to present the structural diversity of triterpene glycoside congeners in the viscera of *H. lessoni*.

These new saponins (Lessoniosides A–G) have the potential to be consumed with applications as dietary supplements, food preservatives (because of their emulsifying and foaming properties), food additives and development of high value products for various industrial applications and as anti-cancer agents.

Our findings demonstrate that the marine world, in particular sea cucumbers, have much to offer human society in the way of nutraceuticals, pharmaceuticals, agrochemicals, cosmeceuticals, and research biochemicals.

## Supplementary Files

Supplementary File 1
