# Supplementary material for: Structure Elucidation of New Acetylated Saponins, Lessoniosides A, B, C, D, and E, and Non-Acetylated Saponins, Lessoniosides F and G, from the Viscera of the Sea Cucumber Holothuria lessoni
Source: Mar Drugs. 2015 Jan 16;13(1):597–617. doi: 10.3390/md13010597 (PMC4306954; doi:10.3390/md13010597)
Supplement: Supplementary File 1 [file marinedrugs-13-00597-s001.pdf]

Supplementary Information

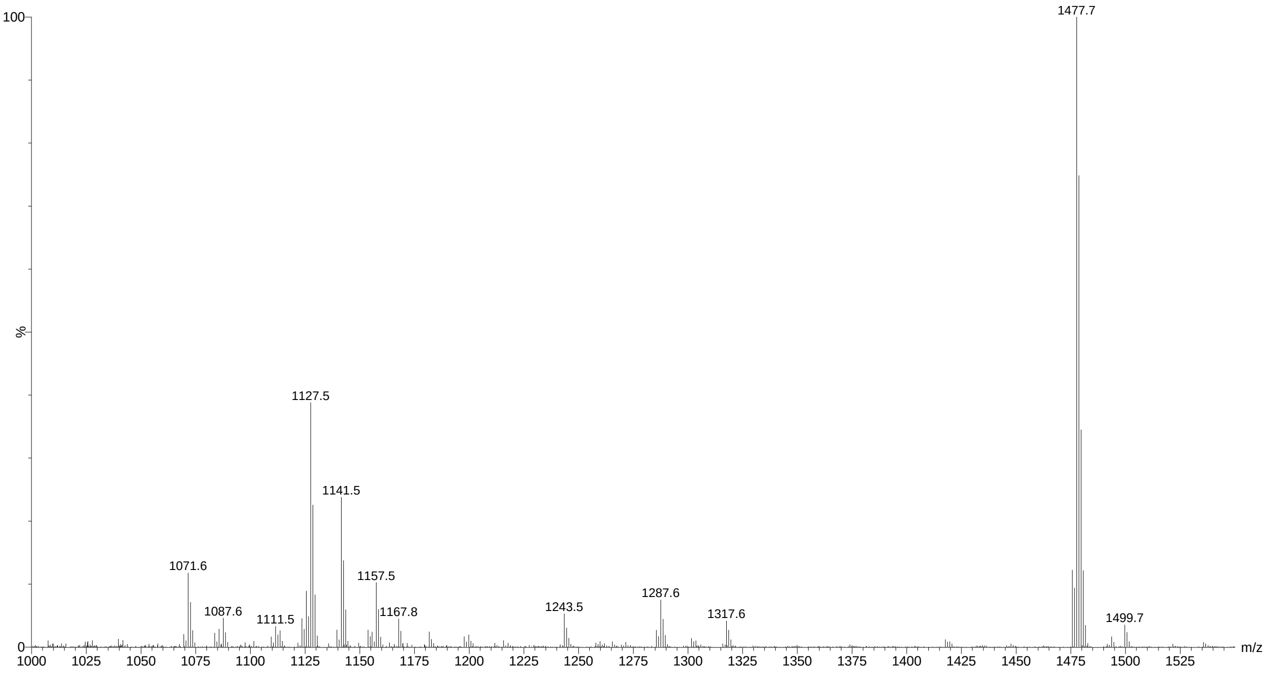

Figure S1. The ESI mass spectrum of HPCPC Fraction 18 from the viscera of the *H. lessoni* in the positive ion mode. A mass range of 1000 to 1530 Da is shown here.

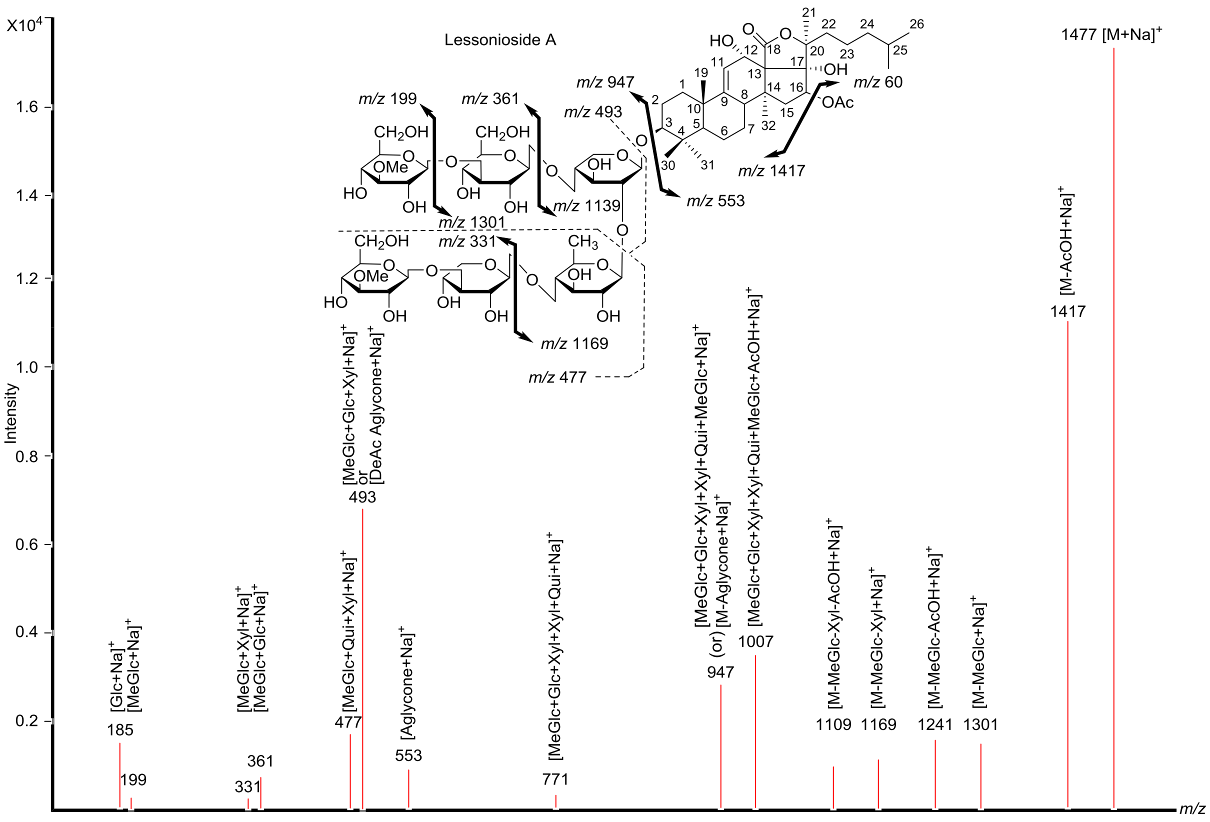

Figure S2. The MALDI MS^2 schematic fragmentation of Lessonioside A in the positive ion mode as a representative.

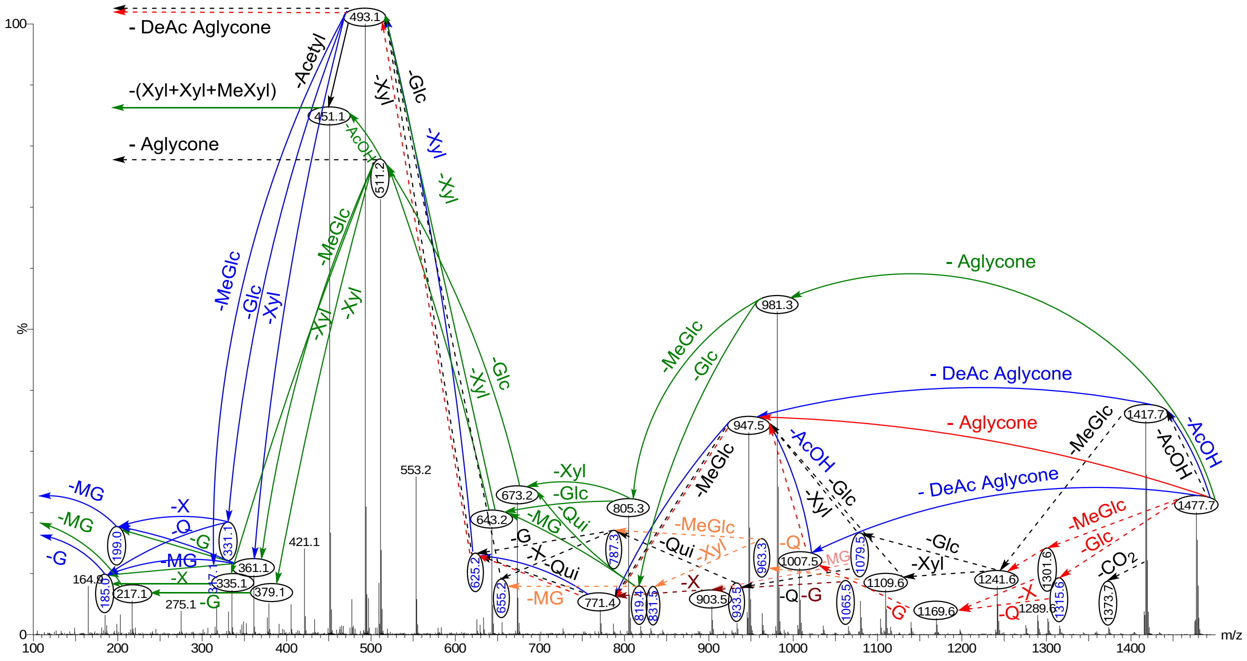

(a)

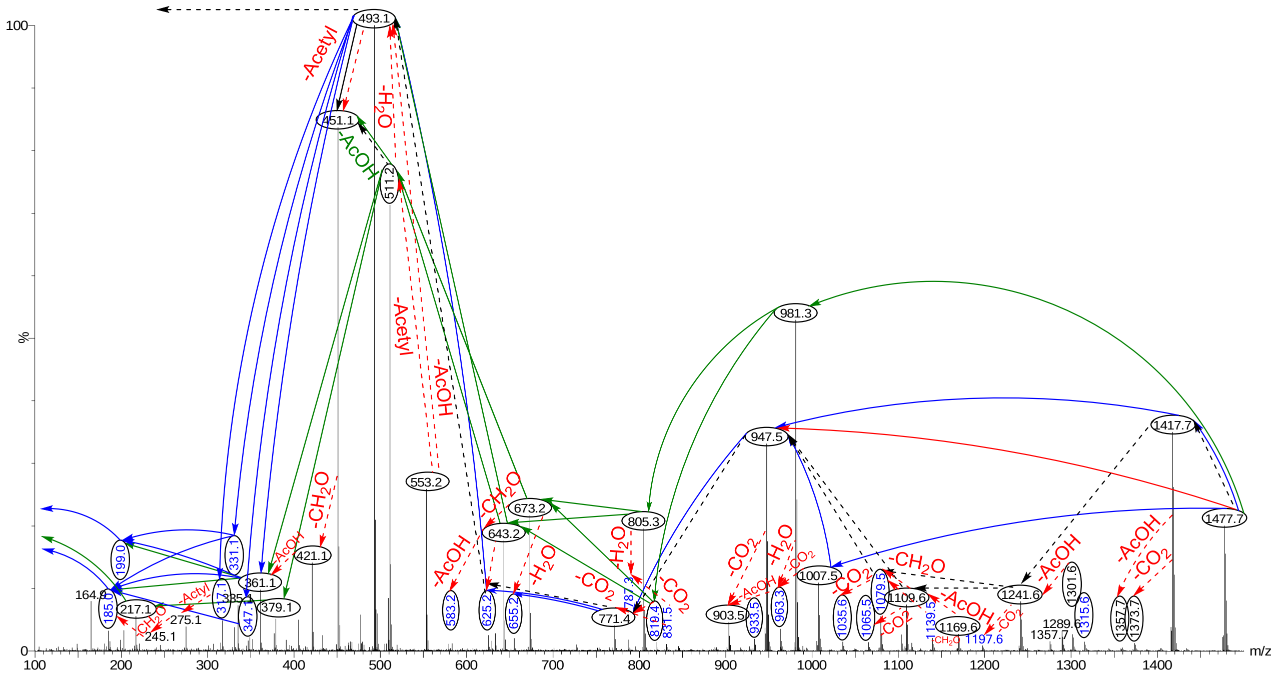

(b)

Figure S3. Ion fragmentation and proposed structures for the isomeric saponins of the precursor ion at *m/z* 1477.7, the complete ESI-MS/MS fragmentation profile (**a**); the losses of neutral molecules (CO_2 and H_2O) (**b**). Full and dotted arrows illustrate the three main feasible fragmentation pathways. The blue arrows indicate the fragmentation of the isomeric congeners Lessoniosides A, B and D where the green arrows show the decomposition patterns of Lessoniosides C, E, F and G. Abbreviations; MG = MeGlc, G = Glc, Q = Qui, X = Xyl, Deacetylated Aglycone = DeAc aglycone.

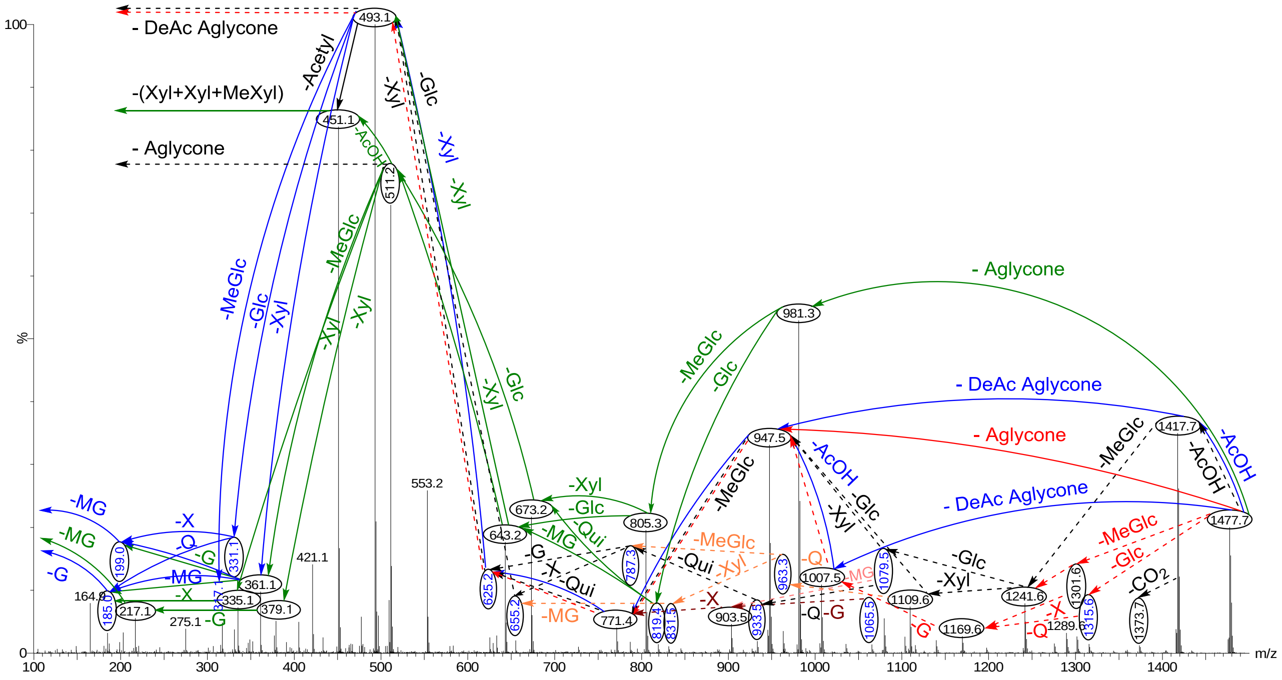

Figure S4. This picture shows the feasible ion fragmentations of the non-acetylated isomeric saponins of the precursor ion at *m/z* 1477.7 from Fraction 18 (green arrows). The losses of Xyl and MeGlc from the ion at *m/z* 511.2 generated the ions at *m/z* 379 and 335, respectively.

© 2015 by the authors; licensee MDPI, Basel, Switzerland. This article is an open access article distributed under the terms and conditions of the Creative Commons Attribution license (http://creativecommons.org/licenses/by/4.0/).
